# Continuous glucose monitoring in adults with type 2 diabetes: a systematic review and meta-analysis

**DOI:** 10.1007/s00125-024-06107-6

**Published:** 2024-02-16

**Authors:** Milena Jancev, Tessa A. C. M. Vissers, Frank L. J. Visseren, Arianne C. van Bon, Erik H. Serné, J. Hans DeVries, Harold W. de Valk, Thomas T. van Sloten

**Affiliations:** 1 Department of Vascular Medicine and Endocrinology, University Medical Center Utrecht, Utrecht, the Netherlands; 2 Department of Internal Medicine, Rijnstate Hospital, Arnhem, the Netherlands; 3 Department of Internal Medicine, Amsterdam University Medical Center, Amsterdam, the Netherlands

**Keywords:** CGM, Continuous glucose monitoring, Glycaemic control, Meta-analysis, Systematic review, Type 2 Diabetes

## Abstract

**Aims/hypothesis:**

Continuous glucose monitoring (CGM) is increasingly used in the treatment of type 2 diabetes, but the effects on glycaemic control are unclear. The aim of this systematic review and meta-analysis is to provide a comprehensive overview of the effect of CGM on glycaemic control in adults with type 2 diabetes.

**Methods:**

We performed a systematic review using Embase, MEDLINE, Web of Science, Scopus and ClinicalTrials.gov from inception until 2 May 2023. We included RCTs investigating real-time CGM (rtCGM) or intermittently scanned CGM (isCGM) compared with self-monitoring of blood glucose (SMBG) in adults with type 2 diabetes. Studies with an intervention duration <6 weeks or investigating professional CGM, a combination of CGM and additional glucose-lowering treatment strategies or GlucoWatch were not eligible. Change in HbA_1c_ and the CGM metrics time in range (TIR), time below range (TBR), time above range (TAR) and glycaemic variability were extracted. We evaluated the risk of bias using the Cochrane risk-of-bias tool version 2. Data were synthesised by performing a meta-analysis. We also explored the effects of CGM on severe hypoglycaemia and micro- and macrovascular complications.

**Results:**

We found 12 RCTs comprising 1248 participants, with eight investigating rtCGM and four isCGM. Compared with SMBG, CGM use (rtCGM or isCGM) led to a mean difference (MD) in HbA_1c_ of −3.43 mmol/mol (−0.31%; 95% CI −4.75, −2.11, *p*<0.00001, *I*^2^=15%; moderate certainty). This effect was comparable in studies that included individuals using insulin with or without oral agents (MD −3.27 mmol/mol [−0.30%]; 95% CI −6.22, −0.31, *p*=0.03, *I*^2^=55%), and individuals using oral agents only (MD −3.22 mmol/mol [−0.29%]; 95% CI −5.39, −1.05, *p*=0.004, *I*^2^=0%). Use of rtCGM showed a trend towards a larger effect (MD −3.95 mmol/mol [−0.36%]; 95% CI −5.46 to −2.44, *p*<0.00001, *I*^2^=0%) than use of isCGM (MD −1.79 mmol/mol [−0.16%]; 95% CI −5.28, 1.69, *p*=0.31, *I*^2^=64%). CGM was also associated with an increase in TIR (+6.36%; 95% CI +2.48, +10.24, *p*=0.001, *I*^2^=9%) and a decrease in TBR (−0.66%; 95% CI −1.21, −0.12, *p*=0.02, *I*^2^=45%), TAR (−5.86%; 95% CI −10.88, −0.84, *p*=0.02, *I*^2^=37%) and glycaemic variability (−1.47%; 95% CI −2.94, −0.01, *p*=0.05, *I*^2^=0%). Three studies reported one or more events of severe hypoglycaemia and macrovascular complications. In comparison with SMBG, CGM use led to a non-statistically significant difference in the incidence of severe hypoglycaemia (RR 0.66, 95% CI 0.15, 3.00, *p*=0.57, *I*^2^=0%) and macrovascular complications (RR 1.54, 95% CI 0.42, 5.72, *p*=0.52, *I*^2^=29%). No trials reported data on microvascular complications.

**Conclusions/interpretation:**

CGM use compared with SMBG is associated with improvements in glycaemic control in adults with type 2 diabetes. However, all studies were open label. In addition, outcome data on incident severe hypoglycaemia and incident microvascular and macrovascular complications were scarce.

**Registration:**

This systematic review was registered on PROSPERO (ID CRD42023418005).

**Graphical Abstract:**

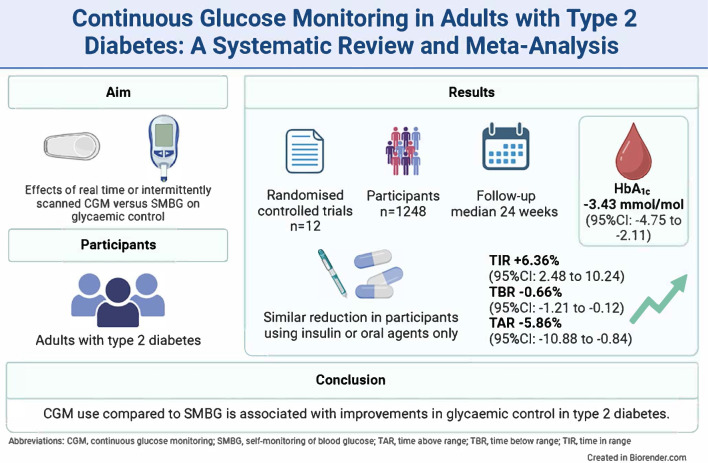

**Supplementary Information:**

The online version of this article (10.1007/s00125-024-06107-6) contains peer-reviewed but unedited supplementary material.


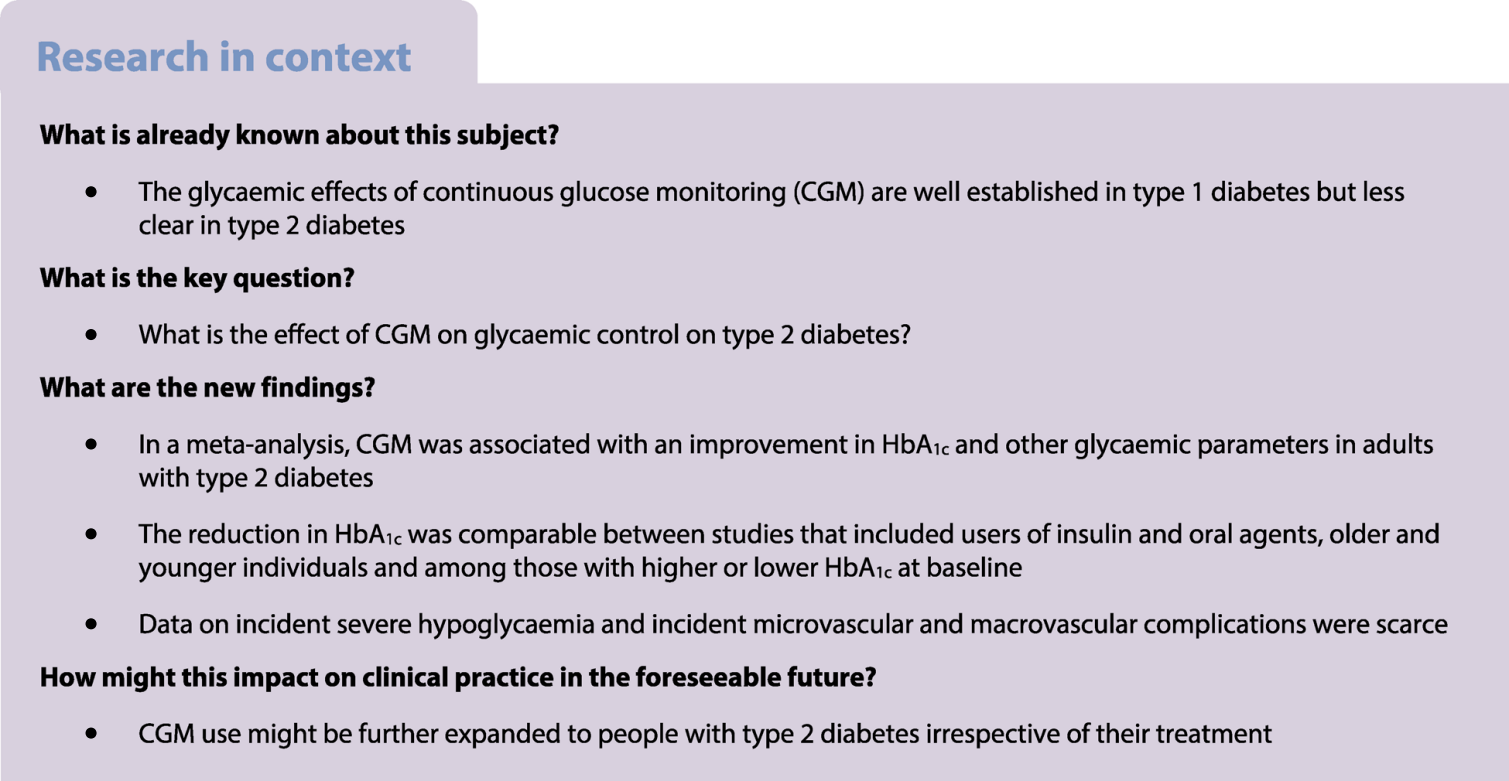


## Introduction

Optimising glycaemic control is a keystone in the management of type 2 diabetes [1]. Fingerstick-based self-monitoring of blood glucose (SMBG) has been the most used method for measuring daily glucose levels [2]. However, this method does not provide continuous data about glucose levels, and, thus, may miss asymptomatic hypo- or hyperglycaemia and does not provide information about the direction of change in glucose levels. Furthermore, SMBG can be painful and increases disease burden [3].

The development of continuous glucose monitoring (CGM), either intermittently scanned CGM (isCGM) systems or real-time CGM (rtCGM) systems, has enabled the monitoring of glucose levels without fingersticks. CGM consists of a subcutaneous sensor that monitors interstitial glucose levels, which approximates blood glucose levels [3]. CGM thereby allows for direct observation of glycaemic excursions and daily glucose profiles that can inform therapy decisions and possibly adjust behaviours [4, 5]. Recent guidelines have recommended CGM use in individuals with type 2 diabetes treated with insulin [1]. However, the extent to which CGM improves glycaemic control in type 2 diabetes is unclear. Furthermore, it is unknown whether any such beneficial effect is different among individuals treated with or without insulin.

Glycaemic control is most often quantified by measurement of HbA_1c_ levels [4], which reflects average glucose over the last 2–3 months. CGM provides additional parameters of glycaemic control, including time in range (TIR), time below range (TBR) and time above range (TAR). These parameters provide information about glucose control on a daily basis, and are increasingly used in clinical research and daily care [1, 4].

To date, there have been seven systematic reviews investigating the effect of CGM on glycaemic control in type 2 diabetes [6–12]. However, these reviews only included a limited number of RCTs, i.e. six studies or fewer, or included studies with a mixed population of both individuals with type 1 diabetes and individuals with type 2 diabetes. Furthermore, previous reviews could not conclude on whether the effect of CGM was different in individuals treated with or without insulin, and most reviews did not investigate the effect of CGM use on the sensor-derived glycaemic parameters TIR, TBR and TAR [8]. Also, no review evaluated the effect on the occurrence of severe hypoglycaemia or development of diabetes-related complications [6].

Therefore, the primary aim of this systematic review and meta-analysis was to give an up-to-date comprehensive overview of the effect of CGM (rtCGM or isCGM) compared with SMBG on glycaemic control, as quantified by HbA_1c_, in adults with type 2 diabetes treated with or without insulin. Secondary aims were to evaluate the effect of CGM use compared with SMBG on TIR, TAR, TBR, glycaemic variability, incident severe hypoglycaemia and incident diabetes-related micro- and macrovascular complications.

## Methods

This systematic review was registered on PROSPERO (ID CRD42023418005) and is reported in accordance with the Preferred Reporting Items for Systematic Reviews and Meta-analysis (PRISMA) statement [13]. The PRISMA checklist is provided in electronic supplementary material (ESM) Table 1.

### Data sources and searches

We searched Embase, MEDLINE (via PubMed), Web of Science, Scopus and ClinicalTrials.gov for relevant articles using a combination of the terms ‘diabetes mellitus type 2’, ‘continuous glucose monitoring’ and ‘HbA_1c_’ from inception until 2 May 2023. Details of the search are provided in ESM Table 2. Additionally, we did a manual search by reviewing the reference lists of all relevant articles identified and prior reviews and meta-analyses to identify any remaining articles. Two reviewers (MJ and TACMV) independently performed screening of titles/abstracts using Rayyan [14], and assessed the full texts for eligibility. Any discrepancies were discussed and resolved by a third reviewer (TTvS).

### Study selection

Studies were eligible if they compared CGM (rtCGM or isCGM) to SMBG (or isCGM when rtCGM was the main intervention) and reported HbA_1c_ as an outcome measure. We included RCTs with a minimum intervention period of 6 weeks of consecutive or intermittent use of CGM among adults with type 2 diabetes (irrespective of diabetes treatment) in an outpatient setting. We excluded studies with pregnant women or individuals with type 1 diabetes, studies that investigated GlucoWatch [15] or a professional CGM (pCGM) device (e.g. Abbott Freestyle Libre Pro IQ or Dexcom G6 Pro) or an intervention that consisted of CGM combined with an additional glucose-lowering treatment strategy.

### Data extraction and quality assessment

Two reviewers (MJ and TACMV) independently extracted data from the included full-text articles using a standardised form. Disagreements were resolved by consensus or a third reviewer (TTvS). We extracted data on the study authors, year of publication, study design and follow-up duration, attrition rate, intervention type and duration, comparator type, inclusion and exclusion criteria, baseline characteristics (age, sex, diabetes duration and ethnicity), baseline insulin use and use of oral glucose-lowering drugs. In addition, we retrieved information on HbA_1c_, TIR, TBR, TAR and glycaemic variability (defined as coefficient of variation [CV]) at baseline and at the endpoint. Finally, data on the incidence of severe hypoglycaemia (as defined in the original publication) and the incidence of microvascular complications (retinopathy, nephropathy and neuropathy) or macrovascular complications (myocardial infarction, cardiovascular death, cerebrovascular disease or peripheral artery disease) at the endpoint. Authors were contacted in case of any missing information.

We used the Cochrane risk-of-bias tool version 2 (RoB 2) to assess risk of bias of the included trials [16]. This tool includes five domains: randomisation process, deviations from intended interventions, missing outcome data, measurement of the outcome and selection of the reported results. The quality of each RCT was assessed independently by two reviewers (MJ and TACMV). Any disagreements were resolved by consensus with a third reviewer (TTvS). Studies were rated as having high, moderate or low risk of bias. We labelled trials as low risk of bias if all five domains were scored as low risk of bias.

### Data synthesis and analysis

The primary outcome was mean difference in HbA_1c_ (% and mmol/mol) from baseline to study end and corresponding 95% CI. Secondary outcomes were TIR (percentage of time glucose was between 3.9–10 mmol/l [70–180 mg/dl]), TBR (percentage of time glucose <3.9 mmol/l [<70 mg/dl]), TAR (percentage of time glucose was >10 mmol/l [>180 mg/dl]), glycaemic variability (CV [%]), incident severe hypoglycaemia (RR and 95% CI), incident microvascular (retinopathy, nephropathy and neuropathy) and macrovascular complications (myocardial infarction, cardiovascular death, cerebrovascular disease and peripheral artery disease). When TIR, TBR or TAR were described as hours and minutes, values were converted to percentage of time. For glycaemic outcomes we extracted the mean change between groups from baseline to endpoint and the SD. Incident severe hypoglycaemia and complications outcomes were analysed as RR and corresponding 95% CI.

A meta-analysis with a pooled estimates and random effects model was performed in Review Manager version 5.4 [17, 18]. A *p* value of <0.05 was considered statistically significant. Heterogeneity was assessed using *I*^2^ and *χ*^2^. *I*^2^ values of 0% to 40%, 30% to 60%, 50% to 90% and 75% to 100% were interpreted as low, moderate, substantial and considerable heterogeneity, respectively [18]. Funnel plots were visually inspected and the Egger test was used to assess publication bias [19]. Grading of Recommendations, Assessment, Development, and Evaluations (GRADE) was used to estimate the certainty of evidence [20].

For the primary outcome, we performed a predefined subgroup analysis considering CGM type (comparing rtCGM to isCGM, isCGM to SMBG, and rtCGM to SMBG). If possible, the main analysis was repeated stratified according to insulin use, median baseline HbA_1c_, median age, median diabetes duration, median intervention duration, the presence of micro- or macrovascular complications at baseline, sex (male vs female) and background glucose-lowering therapy (insulin users, oral agents users only, and mix of insulin and oral agent users).

## Results

### Search results

The initial search resulted in 8000 articles (ESM Fig. 1). After removal of duplicates, 3994 articles were screened for title and abstract, of which 3971 articles were excluded. Articles were mainly excluded due to incorrect study design (e.g. observational study or no relevant research question) (*n*=2111). The full texts of 23 studies were assessed, of which 11 were excluded because of incorrect study design (*n*=5), use of a device other than rtCGM or isCGM (*n*=4), study duplicate (*n*=1) or not studying individuals with type 2 diabetes (*n*=1). Finally, 12 RCTs were included. Corresponding authors of the publications of all 12 RCTs were contacted to obtain any missing data, and the investigators of five RCTs [21–25] provided additional data.

### Characteristics of included trials

The 12 RCTs were published between 2008 and 2023 and included a total of 1248 participants. The sample size ranged from 25 to 224 participants (Table 1). Participants (43.3% female) had a mean age of 58.9 years and a mean diabetes duration of 14.7 years. The baseline HbA_1c_ ranged from a mean of 61.1 mmol/mol (7.83%) to 77.9 mmol/mol (9.27%) (Table 1). Eleven trials had a two-arm open-label parallel group design [21–31]. One trial had a three-arm parallel group design [32], including arms with 1 week of CGM use at baseline only (first arm), 1 week of CGM use at both baseline and at the end of the study period after 12 weeks (second arm) and a control arm that did not use CGM (third arm) [32]. We only included data of the intervention arm with CGM use at both the start and study endpoint and control. Eight trials compared rtCGM to SMBG [22, 26–32] and four trials compared isCGM to SMBG [21, 23–25]. No studies compared rtCGM to isCGM. The intervention duration ranged from 10 to 34 weeks (Table 1). Five trials had an extended follow-up period of 24 to 52 weeks [21, 24, 29, 30, 32]. In seven trials, CGM was used continuously [21–26, 28], whereas five trials used intermittent wearing of the CGM ranging from two cycles of 2 days to continuous use [27, 29–32]. As the primary outcome, 10 trials had HbA_1c_ [22–24, 26–32], one trial had TIR [21] and another trial had treatment satisfaction [25]. Four trials included participants on insulin therapy only [22, 23, 25, 28], three trials included participants on oral glucose-lowering medication only [24, 29, 32] and five trials included participants on both insulin and/or oral glucose-lowering medication [21, 26, 27, 30, 31].

**Table 1:** Characteristics of the RCTs identified

Study (first author, year)	Study design	Intervention duration (weeks)	Study duration (weeks)	*N* intervention/control	Mean age intervention/control (years)	Mean baseline HbA_1c_ intervention/control (mmol/mol)	Mean baseline HbA_1c_ intervention/control (%)	Intervention /sensor type/comparator	Medication use intervention/control (%)	Primary outcome
Ajjan et al (2023) [21]	2-arm RCT	12	43	69/72	63.2/62.0	74.6/77.9	8.97/9.27	isCGM/Freestyle Libre sensor/SMBG	Insulin: 52.2/47.2 SU: 47.8/52.8 Metformin: 72.5/77.8 DPP4-i: 21.7/15.3 SGLT-2i: 10.1/20.8 GLP-1 RA: 7.2/6.9 Thiazolidinedione: 2.9/0.0	TIR
Beck et al (2017) [22]	2-arm RCT	24	24	79/79	60.0/60.0	69/69	8.5/8.5	rtCGM/Dexcom G4 Platinum CGM System/SMBG	Insulin: 100/100 Metformin: 56/52 DPP4-i: 7.6/5.1 SLGT-2i: 13/13 GLP-1 RA: 14/10 Other (not specified): 8.9/13	HbA_1c_
Bergenstal et al (2022) [26]	2-arm RCT	16	16	59/55	59.3/58.8	66.0/62.3	8.19/7.85	rtCGM/Type not specified/SMBG	Insulin, SU, Metformin, DPP-4i, GLP-1 RA (% not specified)	HbA_1c_
Cosson et al (2009) [27]	2-arm RCT	12 (2 days at baseline, 2 days at week 12)	12	11/14	57.2/57.3	77.3/75.6	9.22/9.07	rtCGM/GlucoDay System/SMBG	Insulin: 27/43 Oral agents (not specified): 73/57	HbA_1c_
Haak et al (2017) [23]	2-arm RCT	24	24	149/75	59.0/59.5	71.0/72.1	8.65/8.75	isCGM/Freestyle Libre sensor/SMBG	Insulin: 100/100	HbA_1c_
Martens et al (2021) [28]	2-arm RCT	34	34	116/59	56.0/59.0	77/75	9.2/9.0	rtCGM/Dexcom G6 CGM System/SMBG	Insulin: 100/100 SU: 36.2/47.5 Metformin: 79.3/78.0 DPP4-i: 4./13.6 SLGT-2i: 9.5/8.5 GLP-1 RA: 23.3/13.6	HbA_1c_
Moon et al (2023) [32]	3-arm RCT	12 (1 week at baseline, 1 week at week 12)	24	15/15	53.9/50.7	66/65	8.2/8.1	rtCGM/Guardian 3 sensor (Medtronic MiniMed)/SMBG	Insulin: 0/0 SU: 73.3/40.0 Metformin: 100/100 DPP4-i: 80.0/86.7 SLGT-2i: 26.7/13.3 Thiazolidinedione: 40.0/66.7	HbA_1c_
Price et al (2021) [29]	2-arm RCT	12 (10 days each at weeks 0, 4 and 8)	36	46/24	58.9/60.9	68/69	8.4/8.5	rtCGM/Dexcom G6 CGM System/SMBG	Insulin: 0/0 SU: 75.6/56.5 DPP4-i: 28.9/43.5 SGLT-2i: 57.8/39.1 Thiazolidinedione: 11.1/13.0 Biguanide: 82.2/82.6 Meglitinide: 0.0/4.3 Other (not specified): 35.6/34.8	HbA_1c_
Vigersky et al (2012) [30]	2-arm RCT	12 (four cycles of 2 weeks/1 week off)	52	50/50	55.5/60.0	68/66	8.4/8.2	rtCGM/Dexcom SEVEN/SMBG	Insulin: 38/28 Oral agents only: 48.0/54.0 Oral agents/exenatide: 8.0/10.0 Diet and exercise only: 6.0/8.0	HbA_1c_
Wada et al (2020) [24]	2-arm RCT	12	24	49/51	58.1/58.7	61.1/62.3	7.83/7.85	isCGM/Free Style Libre System/SMBG	Insulin: 0/0 SU: 32.7/27.5 Metformin: 69.4/62.7 DPP4-i: 81.6/78.4 SGLT-2i: 42.9/37.3 GLP-1 RA: 2.0/5.9 Glinide: 20.4/21.6 α-Glucosidase-i: 26.5/35.3 Pioglitazone: 8.2/13.7	HbA_1c_
Yaron et al (2019) [25]	2-arm RCT	10	10	53/48	67.6/65.9	71.4/67.7	8.68/8.34	isCGM/Free Style Libre System/SMBG	Insulin: 100/100 SU: 0.0/4.2 Metformin: 71.7/72.9 DPP4-i: 7.5/14.6 SGLT-2i: 24.5/27.7 GLP-1 RA: 35.8/31.3	Treatment satisfaction
Yoo et al (2008) [31]	2-arm RCT	12 (once a month for 3 days)	12	32/33	54.6/57.5	76/72	9.1/8.7	rtCGM/Guardian RT CGM System/SMBG	Insulin: 13.8/17.9 Oral agents only: 44.8/35.7 Combination of insulin and oral medications: 37.9/42.9	HbA_1c_

DPP4-i, dipeptidyl peptidase-4 inhibitor; GLP-1RA, glucagon-like peptide-1 receptor agonists; α-Glucosidase-i, α-glucosidase inhibitor; SGLT-2i, sodium–glucose cotransporter 2 inhibitor; SU, sulfonylurea

### Risk of bias

Four trials [21, 22, 24, 28] had an overall low risk of bias, whereas eight trials [23, 25–27, 29–32] had some concerns (ESM Fig. 2). As for the domain of ‘randomisation process’, three trials [25, 29, 30] were graded with some concerns due to lack of information about the randomisation and allocation concealment process. All trials were graded with low risk of bias for the domains ‘deviation from the intended interventions’, ‘missing outcome data’ and ‘measurement of the outcome’. As for the domain of ‘selection of the reported results’, seven trials [23, 25–27, 29, 31, 32] were graded with some concerns due to missing trial protocols and statistical analysis plan.

### Primary outcome

**Fig. 1:**
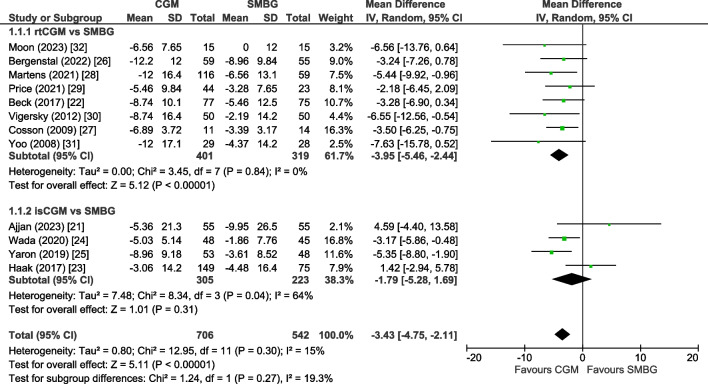
Forest plot of pooled analysis of change in HbA_1c_ (mmol/mol) in individuals with type 2 diabetes using rtCGM or isCGM compared with SMBG

Results of all included 12 trials [21–32] were pooled in the primary meta-analysis. The mean change in HbA_1c_ level was −3.43 mmol/mol (−0.31%; 95% CI −4.75, −2.11, *p*<0.00001) in favour of the CGM group (Fig. 1). Heterogeneity was low (*I*^2^=15%) and we found no evidence of publication bias (ESM Fig. 3; Egger test, *p*=0.29). Using GRADE criteria, the HbA_1c_ outcome was graded with moderate certainty (Table 2).

**Table 2:** Determining the certainty of evidence of outcomes using GRADE criteria

Variable	Certainty assessment							Number of patients		Effect		Certainty
	Number of studies	Study design	Risk of bias	Inconsistency	Indirectness	Imprecision	Other considerations	CGM	SMBG	RR (95% CI)	Absolute (95% CI)	
HbA_1c_	12	RCT	Not serious	Not serious	Serious	Not serious	None	706	542	-	MD 3.43 lower (4.75 lower, 2.11 lower)	⨁⨁⨁◯ Moderate
TIR	8	RCT	Not serious	Not serious	Serious	Not serious	None	554	383	-	MD 6.36 higher (2.48 higher, 10.24 higher)	⨁⨁⨁◯ Moderate
TBR	9	RCT	Not serious	Serious	Serious	Not serious	None	561	395	-	MD 0.66 lower (1.21 lower, 0.12 lower)	⨁⨁◯◯ Low
TAR	8	RCT	Not serious	Not serious	Serious	Not serious	None	554	383	-	MD 5.86 lower (10.88 lower, 0.84 lower)	⨁⨁⨁◯ Moderate
Glycaemic variability	5	RCT	Not serious	Not serious	Serious	Serious	None	399	259	-	MD 1.47 lower (2.94 lower, 0.01 lower)	⨁⨁◯◯ Low
Severe hypoglycaemia	9	RCT	Not serious	Not serious	Not serious	Very serious	None	4/334 (1.2%)	4/206 (1.9%)	RR 0.66 (0.15, 3.00)	3 fewer per 1.000 (8 fewer, 8 more)	⨁⨁◯◯ Low
Macrovascular complications	3	RCT	Not serious	Serious	Not serious	Very serious	None	21/190 (11.1%)	12/170 (7.1%)	RR 1.54 (0.42, 5.72)	38 more per 1.000 (41 fewer, 333 more)	⨁◯◯◯ Very low

MD, mean difference

### Subgroup analysis

In the eight trials on rtCGM (*n*=720) [22, 26–32], the pooled mean change in HbA_1c_ level was −3.95 mmol/mol (−0.36%) (95% CI −5.46, −2.44, *p*<0.00001, *I*^2^=0%) in favour of the rtCGM group. In the four trials on isCGM (*n*=528) [21, 23–25], we found a non-significant mean change in HbA_1c_ level of −1.79 mmol/mol (−0.16%; 95% CI −5.28, 1.69, *p*=0.31) in favour of the isCGM group with substantial heterogeneity (*I*^2^=64%).

**Fig. 2:**
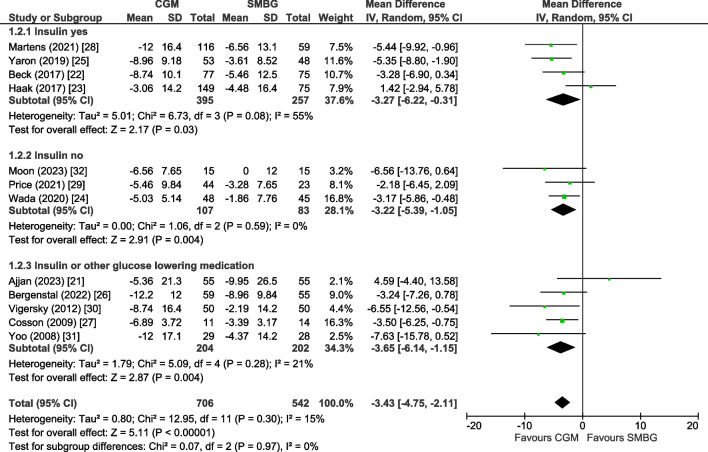
Forest plot of pooled analysis of change in HbA_1c_ (mmol/mol) in individuals with type 2 diabetes using rtCGM or isCGM compared with SMBG, stratified according to type of glucose-lowering therapy (insulin users, no insulin users or mixed population of insulin users and no insulin users)

Trials including participants all using insulin [22, 23, 25, 28] showed a mean change in HbA_1c_ level of −3.27 mmol/mol (−0.30%; 95% CI −6.22, −0.31, *p*=0.03, *I*^2^=55%), trials including users of oral agents only [24, 29, 32] showed a mean change of −3.22 mmol/mol (−0.29%; 95% CI −5.39, −1.05, *p*=0.004, *I*^2^=0%) and studies including users of both insulin or oral agents [21, 26, 27, 30, 31] showed a mean change of −3.65 mmol/mol (−0.33%; 95% CI −6.14, −1.15, *p*=0.004, *I*^2^=21%) (Fig. 2). Subgroup analyses for baseline HbA_1c_, age, diabetes duration and intervention duration showed comparable change in HbA_1c_ level in each subgroup (ESM Fig. 4–7). Subgroup analyses for sex, the presence of microvascular and macrovascular complications or background glucose-lowering therapy other than insulin at baseline were not possible due to lack of data.

### Secondary outcomes

**Fig. 3:**
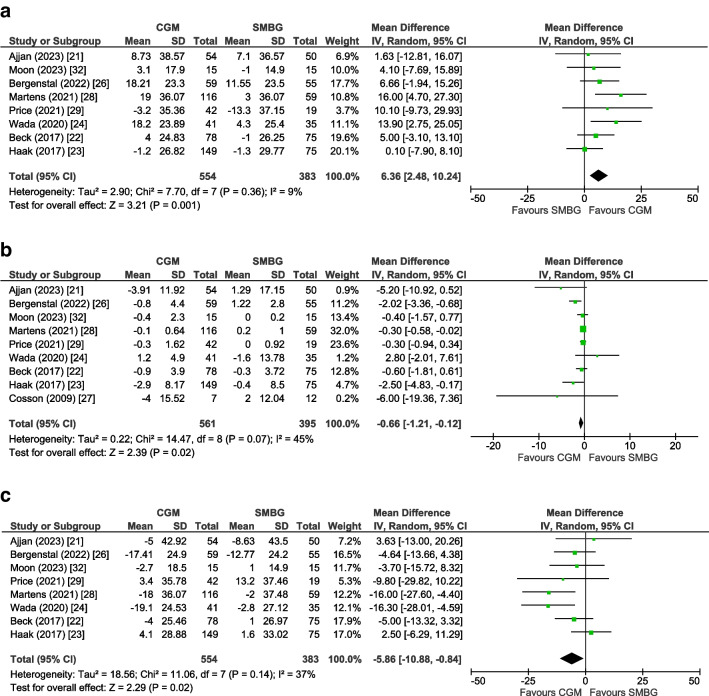
Forest plot of pooled analysis of change in TIR (**a**) TBR (**b**) and TAR (**c**) in individuals with type 2 diabetes using rtCGM or isCGM compared with SMBG

For TIR and TAR as the outcome, eight trials (*n*=937 participants) [21–24, 26, 28, 29, 32] could be included in a pooled analysis, for TBR nine trials (*n*=956 participants) [21–24, 26–29, 32], and for glycaemic variability (CV) five trials (*n*=658 participants) [22–24, 28, 32]. The mean change for TIR was +6.36% (95% CI +2.48, +10.24, *p*=0.001, *I*^2^=9%), for TBR −0.66% (95% CI −1.21, −0.12, *p*=0.02, *I*^2^=45%), for TAR −5.86% (95% CI −10.88, −0.84, *p*=0.02, *I*^2^=37%) (Fig. 3) and for glycaemic variability −1.47% (95% CI −2.94, −0.01, *p*=0.05, *I*^2^=0%), all in favour of the CGM group (ESM Fig. 8).

Nine studies [21–25, 28, 29, 31, 32] reported on severe hypoglycaemia, yet only three studies [21, 23, 28] reported one or more events in one of the groups and contributed to the meta-analysis. There was no statistically significant difference in the incidence of severe hypoglycaemia (RR 0.66, 95% CI 0.15, 3.00, *p*=0.57, *I*^2^=0%). In addition, based on three trials [21, 23, 28], there was no statistically significant difference in the incidence of macrovascular complications (RR 1.54, 95% CI 0.42, 5.72, *p*=0.52, *I*^2^=29%) in the CGM group compared with SMBG (ESM Fig. 9–10). No trials reported outcome data on microvascular complications.

Using GRADE criteria, the outcomes TIR and TAR were graded with moderate certainty. The outcomes TBR, glycaemic variability and severe hypoglycaemia were graded with low certainty, whereas outcome macrovascular complications was graded with very low certainty. Outcomes were downgraded partly because of inconsistency and the low number of events resulting in imprecision (Table 2).

## Discussion

This systematic review and meta-analysis on the effect of CGM use (rtCGM or isCGM) on glycaemic control in adults with type 2 diabetes showed a modest reduction of −3.43 mmol/mol (−0.31%) in HbA_1c_. This effect was comparable among users of insulin and other oral agents. Furthermore, CGM was associated with a +6.36% increase in TIR and a decrease of −0.66% in TBR, −5.86% in TAR and −1.47% in glycaemic variability.

Our results are in accordance with previous systematic reviews and meta-analyses, which found a significant reduction in HbA_1c_ ranging from −7.65 mmol/mol (−0.70%) to −2.73 mmol/mol (−0.25%) in individuals with type 2 diabetes [6–10, 12]. Moreover, our result is comparable with a systematic review showing a reduction of −2.46 mmol/mol (0.23%) in HbA_1c_ with CGM use compared with SMBG in individuals with type 1 diabetes [33]. Our review extends previous reviews [6–11] because we could include an additional six RCTs including 589 participants, which allowed us to calculate a more precise effect size with higher statistical power. This also allowed us to evaluate the effect of CGM use in users of insulin and oral agents, according to CGM type (rtCGM and isCGM) and in relevant subgroups.

We found a reduction in HbA_1c_ that was comparable between studies including both users of insulin and other oral agents. Current guidelines suggest CGM as a therapy strategy only in individuals with type 2 diabetes who use insulin [1]. Our findings, however, might support the efficacy of CGM in individuals with type 2 diabetes, irrespective of glucose-lowering therapy. CGM use may improve dosing of any glucose-lowering therapy (insulin and other oral agents) and/or stimulate a healthy lifestyle, and this may explain its beneficial effects on glycaemic control compared with SMBG [1].

In our analyses, we found a trend towards a larger reduction in HbA_1c_ in studies investigating rtCGM rather than those investigating isCGM. This is in accordance with a previous systematic review that reported, in a subgroup analysis, a non-significant change in HbA_1c_ in both type 1 diabetes or type 2 diabetes [11]. This finding might suggest that real-time techniques might provide additional benefit compared with techniques requiring intermittent scanning. However, no study directly compared rtCGM to isCGM in type 2 diabetes and this issue, therefore, requires further study.

The mean reduction in HbA_1c_ of −3.43 mmol/mol (−0.31%) was relatively modest, but the effect was consistent across all included studies, indicating the robustness of the study findings. Furthermore, the effect was consistent across studies with younger and older individuals, short and long diabetes duration and higher or lower HbA_1c_ at baseline. Also, consistent beneficial effects of CGM were found on other markers of glycaemic control, i.e. TIR, TBR, TAR and glycaemic variability [1]. The beneficial effect on TIR (+6.36%) was more than the current consensus of 5% minimal clinical relevant difference in TIR [34].

We found a non-significant decrease in the incidence of severe hypoglycaemia. This was, however, based on only three studies with a small number of events (eight events in total). Most studies that assessed severe hypoglycaemia reported no events in both groups. Therefore, we likely had insufficient power to detect a difference in incidence of severe hypoglycaemia. Furthermore, only three trials assessed macrovascular complications, with most events detected in one study [21]. This study was performed in people who had experienced a recent myocardial infarction. Thus, our aggregated results for macrovascular complications have limited generalisability and should be interpreted with caution. A reduction in HbA_1c_ would likely translate to lower rates of diabetes-related complications in the long term, but this cannot be substantiated in this data.

Strengths of our analysis include the comprehensive overview of the effect of both rtCGM and isCGM on glycaemic control in adults with type 2 diabetes with different glucose-lowering therapies, and the analysis of multiple relevant glycaemic outcome parameters. In addition, our study findings are consistent with, and extend the results of, a recent meta-analysis [12]. We did a more recent search and identified one additional RCT [21]. Furthermore, we were able to do a large range of prespecified subgroup analyses. Limitations include the fact that we did not include quality of life as outcome. However, previous trials have demonstrated that CGM use in type 2 diabetes is associated with beneficial effects on quality of life compared with SMBG [25, 32]. In contrast to studies done in individuals with type 1 diabetes [33] we did not find different effects of glucose sensor use according to baseline HbA_1c_ levels. However, we may not have had sufficient power to detect any differences related to baseline HbA_1c_, because all studies except one [24] had a baseline HbA_1c_ value of 64 mmol/mol (8%) or higher. In addition, all RCTs were open label, the study duration of included RCTs was relatively short (maximum 52 weeks) and no or only limited data were available on incident severe hypoglycaemia and incident microvascular and macrovascular complications.

In conclusion, this systematic review and meta-analysis showed an improvement in HbA_1c_ and other parameters of glycaemic control related to CGM use (rtCGM or isCGM) in adults with type 2 diabetes. Future studies are needed to compare the effect on glycaemic control of rtCGM to isCGM and assess the effect of CGM use on incident micro- and macrovascular complications.

### Supplementary Information

Below is the link to the electronic supplementary material.

Supplementary file1 (PDF 337 KB)

## Data Availability

Data are available from the corresponding author upon request.

## Abbreviations

CGM: Continuous glucose monitoring
CV: Coefficient of variation
GRADE: Grading of Recommendations, Assessment, Development, and Evaluations
isCGM: Intermittently scanned continuous glucose monitoring
PRISMA: Preferred Reporting Items for Systematic Reviews and Meta-analysis
SMBG: Self-monitoring of blood glucose
rtCGM: Real-time continuous glucose monitoring
TAR: Time above range
TBR: Time below range
TIR: Time in range
