# Supplementary material for: Continuous glucose monitoring in adults with type 2 diabetes: a systematic review and meta-analysis
Source: Diabetologia. 2024 Feb 16;67(5):798–810. doi: 10.1007/s00125-024-06107-6 (PMC10954850; doi:10.1007/s00125-024-06107-6)

Supplementary Data

Index

ESM Table 1. PRISMA (2020) Checklist 2

ESM Table 2. Search terms details. 5

ESM Fig. 1 Search flow and study selection. 7

ESM Fig. 2 Risk of bias in the included studies 8

ESM Fig. 3 Funnel plot of the main analysis (change in HbA1c). 9

ESM Fig. 4 Forest plot of change in HbA1c (mmol/mol) in individuals with type 2 diabetes using real time or intermittently scanning continuous glucose monitoring compared to self-monitoring of blood glucose; results according to median baseline HbA1c (69 mmol/mol). 10

ESM Fig. 5 Forest plot of change in HbA1c (mmol/mol) in individuals with type 2 diabetes using real time or intermittently scanning continuous glucose monitoring compared to self-monitoring of blood glucose; results 11

according to median baseline age (58.9 years). 11

ESM Fig. 6 Forest plot of change in HbA1c (mmol/mol) in individuals with type 2 diabetes using real time or intermittently scanning continuous glucose monitoring compared to self-monitoring of blood glucose; results according to median diabetes duration (13.4 years). 12

ESM Fig. 8 Forest plot of change in glycemic variability (coefficient of variation (%)) in individuals with type 2 diabetes using real time or intermittently scanning continuous glucose monitoring compared to self-monitoring of blood glucose 14

ESM Fig. 9 Forest plot of change in the incident risk of severe hypoglycemia in individuals with type 2 diabetes using real time or intermittently scanning continuous glucose monitoring compared to self-monitoring of blood glucose. 14

ESM Fig. 10 Forest plot of change in the incident risk on macrovascular complications in individuals with type 2 diabetes using real time or intermittently scanning continuous glucose monitoring compared to self-monitoring of blood glucose 15

ESM Table 1. PRISMA (2020) Checklist

Section and Topic	Item #	Checklist item	Location where item is reported
**TITLE**			
Title	1	Identify the report as a systematic review.	p.1
**ABSTRACT**			
Abstract	2	See the PRISMA 2020 for Abstracts checklist.	p.2
**INTRODUCTION**			
Rationale	3	Describe the rationale for the review in the context of existing knowledge.	p.5-6
Objectives	4	Provide an explicit statement of the objective(s) or question(s) the review addresses.	p.6
**METHODS**			
Eligibility criteria	5	Specify the inclusion and exclusion criteria for the review and how studies were grouped for the syntheses.	p.7
Information sources	6	Specify all databases, registers, websites, organisations, reference lists and other sources searched or consulted to identify studies. Specify the date when each source was last searched or consulted.	p.6-7
Search strategy	7	Present the full search strategies for all databases, registers and websites, including any filters and limits used.	p.7
Selection process	8	Specify the methods used to decide whether a study met the inclusion criteria of the review, including how many reviewers screened each record and each report retrieved, whether they worked independently, and if applicable, details of automation tools used in the process.	p.7
Data collection process	9	Specify the methods used to collect data from reports, including how many reviewers collected data from each report, whether they worked independently, any processes for obtaining or confirming data from study investigators, and if applicable, details of automation tools used in the process.	p.8
Data items	10a	List and define all outcomes for which data were sought. Specify whether all results that were compatible with each outcome domain in each study were sought (e.g. for all measures, time points, analyses), and if not, the methods used to decide which results to collect.	p.8-9
	10b	List and define all other variables for which data were sought (e.g. participant and intervention characteristics, funding sources). Describe any assumptions made about any missing or unclear information.	p.9
Study risk of bias assessment	11	Specify the methods used to assess risk of bias in the included studies, including details of the tool(s) used, how many reviewers assessed each study and whether they worked independently, and if applicable, details of automation tools used in the process.	p.8
Effect measures	12	Specify for each outcome the effect measure(s) (e.g. risk ratio, mean difference) used in the synthesis or presentation of results.	p.8-9
Synthesis methods	13a	Describe the processes used to decide which studies were eligible for each synthesis (e.g. tabulating the study intervention characteristics and comparing against the planned groups for each synthesis (item #5)).	p.8-9
	13b	Describe any methods required to prepare the data for presentation or synthesis, such as handling of missing summary statistics, or data conversions.	p.8-9

Section and Topic	Item #	Checklist item	Location where item is reported
	13c	Describe any methods used to tabulate or visually display results of individual studies and syntheses.	p.9
	13d	Describe any methods used to synthesize results and provide a rationale for the choice(s). If meta-analysis was performed, describe the model(s), method(s) to identify the presence and extent of statistical heterogeneity, and software package(s) used.	p.9
	13e	Describe any methods used to explore possible causes of heterogeneity among study results (e.g. subgroup analysis, meta-regression).	p.9
	13f	Describe any sensitivity analyses conducted to assess robustness of the synthesized results.	p.9
Reporting bias assessment	14	Describe any methods used to assess risk of bias due to missing results in a synthesis (arising from reporting biases).	p.8-9
Certainty assessment	15	Describe any methods used to assess certainty (or confidence) in the body of evidence for an outcome.	p.9
**RESULTS**			
Study selection	16a	Describe the results of the search and selection process, from the number of records identified in the search to the number of studies included in the review, ideally using a flow diagram.	p.9-10, Fig. 1
	16b	Cite studies that might appear to meet the inclusion criteria, but which were excluded, and explain why they were excluded.	p.9-10
Study characteristics	17	Cite each included study and present its characteristics.	p.10
Risk of bias in studies	18	Present assessments of risk of bias for each included study.	p.11
Results of individual studies	19	For all outcomes, present, for each study: (a) summary statistics for each group (where appropriate) and (b) an effect estimate and its precision (e.g. confidence/credible interval), ideally using structured tables or plots.	p.11-13
Results of syntheses	20a	For each synthesis, briefly summarise the characteristics and risk of bias among contributing studies.	p.11
	20b	Present results of all statistical syntheses conducted. If meta-analysis was done, present for each the summary estimate and its precision (e.g. confidence/credible interval) and measures of statistical heterogeneity. If comparing groups, describe the direction of the effect.	p.11-13
	20c	Present results of all investigations of possible causes of heterogeneity among study results.	p.11-13
	20d	Present results of all sensitivity analyses conducted to assess the robustness of the synthesized results.	p.11-13
Reporting biases	21	Present assessments of risk of bias due to missing results (arising from reporting biases) for each synthesis assessed.	p.11-13
Certainty of evidence	22	Present assessments of certainty (or confidence) in the body of evidence for each outcome assessed.	p.13
**DISCUSSION**			
Discussion	23a	Provide a general interpretation of the results in the context of other evidence.	p.13-14
	23b	Discuss any limitations of the evidence included in the review.	p.15
	23c	Discuss any limitations of the review processes used.	p.15
	23d	Discuss implications of the results for practice, policy, and future research.	p.14-16
**OTHER INFORMATION**			

Section and Topic	Item #	Checklist item	Location where item is reported
Registration and protocol	24a	Provide registration information for the review, including register name and registration number, or state that the review was not registered.	p.6
	24b	Indicate where the review protocol can be accessed, or state that a protocol was not prepared.	p.6
	24c	Describe and explain any amendments to information provided at registration or in the protocol.	p.6
Support	25	Describe sources of financial or non-financial support for the review, and the role of the funders or sponsors in the review.	p.16
Competing interests	26	Declare any competing interests of review authors.	p.16
Availability of data, code and other materials	27	Report which of the following are publicly available and where they can be found: template data collection forms; data extracted from included studies; data used for all analyses; analytic code; any other materials used in the review.	p.6

ESM Table 2. Search terms details.

Database	Search terms
PubMed/MEDLINE	("diabetes mellitus, type 2"[MeSH Terms] OR "type 2 diabetes"[Title/Abstract] OR "diabetes mellitus type ii"[Title/Abstract] OR "diabetes type 2"[Title/Abstract])<br>AND<br>("continuous glucose monitoring"[Title/Abstract:~2] OR "cgm"[Title/Abstract] OR "continuous monitor"[Title/Abstract] OR "real time continuous glucose monitor"[Title/Abstract] OR "rt cgm"[Title/Abstract] OR "rtcgm"[Title/Abstract] OR "is cgm"[Title/Abstract] OR "iscgm"[Title/Abstract] OR "intermittently continuous glucose monitoring"[Title/Abstract:~2] OR "intermittent continuous glucose monitoring"[Title/Abstract:~2] OR "fgm"[Title/Abstract] OR "DexCom"[Title/Abstract] OR "guardian"[Title/Abstract] OR "Paradigm"[Title/Abstract] OR "Medtronic"[Title/Abstract] OR "freestyle"[Title/Abstract] OR "flash glucose monitoring"[Title/Abstract:~2])<br>AND<br>("Glycemic control"[Title/Abstract] OR "Glycaemic control"[Title/Abstract] OR "glycated hemoglobin"[MeSH Terms] OR "glycemic outcome*"[Title/Abstract] OR "glycaemic outcome*"[Title/Abstract] OR "HbA1c"[Title/Abstract] OR "glycemia"[Title/Abstract] OR "glycaemia"[Title/Abstract] OR "time in range"[Title/Abstract] OR "glucose"[Title/Abstract])
Embase	('diabetes mellitus type 2':ti,ab OR 'type 2 diabetes':ti,ab OR 'diabetes type 2':ti,ab)<br>AND<br>('continuous glucose monitoring system'/exp OR 'continuous glucose monitoring system' OR 'cgm':ti,ab OR 'real time continuous glucose monitor*':ti,ab OR 'rt cgm':ti,ab OR 'rtcgm':ti,ab OR 'is cgm':ti,ab OR 'iscgm':ti,ab OR 'intermittently continuous glucose monitoring':ti,ab OR 'intermittent continuous glucose monitor*':ti,ab OR 'fgm':ti,ab OR 'flash glucose monitor*':ti,ab OR 'dexcom':ti,ab OR 'guardian':ti,ab OR 'paradigm':ti,ab OR 'medtronic':ti,ab OR 'freestyle':ti,ab)<br>AND<br>('hemoglobin a1c'/exp OR 'hemoglobin a1c' OR 'glycemic control':ti,ab OR 'glycaemic outcome*':ti,ab OR 'glycemia':ti,ab OR 'glycaemia':ti,ab OR 'time in range':ti,ab OR 'hba1c':ti,ab OR 'glucose':ti,ab)
Scopus	TITLE-ABS("Diabetes Mellitus type 2") OR TITLE-ABS("Diabetes type 2") OR TITLE-ABS("Type 2 diabetes") OR TITLE-ABS("Diabetes mellitus type II")<br>AND<br>TITLE-ABS("Continuous glucose monitor*") OR TITLE-ABS("CGM") OR TITLE-ABS("Continuous monitor*") OR TITLE-ABS("Real time continuous glucose monitor*") OR TITLE-ABS("rt cgm") OR TITLE-ABS("rtcgm") OR TITLE-ABS("is cgm") OR TITLE-ABS("iscgm") OR TITLE-ABS("intermittently continuous glucose monitor*") OR TITLE-ABS("intermittent continuous glucose monitor*") OR TITLE-ABS("FGM") OR TITLE-ABS("Flash glucose monitor*") OR TITLE-ABS("DexCom") OR TITLE-ABS("guardian") OR TITLE-ABS("Paradigm") OR TITLE-ABS("Medtronic") OR TITLE-ABS("Freestyle")<br>AND<br>TITLE-ABS("Glycemic control") OR TITLE-ABS("Glycaemic control") OR TITLE-ABS("Glycated hemoglobin") OR TITLE-ABS("Glycemic outcome*") OR TITLE-ABS("Glycaemic outcome*") OR TITLE-ABS("Glycemia") OR TITLE-ABS("Glycaemia") OR

	TITLE-ABS("Time in Range") OR TITLE-ABS("HbA1c") OR TITLE-ABS("Glucose")
Web of Science	(TS=(Diabetes Mellitus type 2) OR TS=(diabetes mellitus type ii) OR TS=(diabetes type 2)) NOT TS=(diabetes mellitus type 1) NOT TS=(diabetes type 1) NOT TS=(type 1 diabetes)<br>AND<br>TS=(Continuous glucose monitor*) OR TS=(CGM) OR TS=(Continuous monitor*) OR TS=(real time continuous glucose monitor*) OR TS=(rt cgm) OR TS=(rtcgm) OR TS=(is cgm) OR TS=(iscgm) OR TS=(intermittently continuous glucose monitor*) OR TS=(intermittent continuous glucose monitor*) OR TS=(fgm) OR TS=(DexCom) OR TS=(guardian) OR TS=(Paradigm) OR TS=(Medtronic) OR TS=(Freestyle) OR TS=(flash glucose monitor*)<br>AND<br>TS=(Glycemic control) OR TS=(Glycaemic control) OR TS=(Glycated hemoglobin) OR TS=(Glycemic outcome*) OR TS=(Glycaemic outcome*) OR TS=(Glycemia) OR TS=(Glycaemia) OR TS=(time in range) OR TS=(HbA1c) OR TS=(glucose)
ClinicialTrials.gov	Condition or disease: “Type 2 diabetes”, Other terms: “Continuous glucose monitoring”, Status recruitment: “Completed”, Age groups: “Adults (18-64 years) and Older adults (65+ years), Sex: “All”, Study type: “Interventional”, Study results: “With results”, Study documents: “Study protocols”.

ESM Fig. 1 Search flow and study selection.

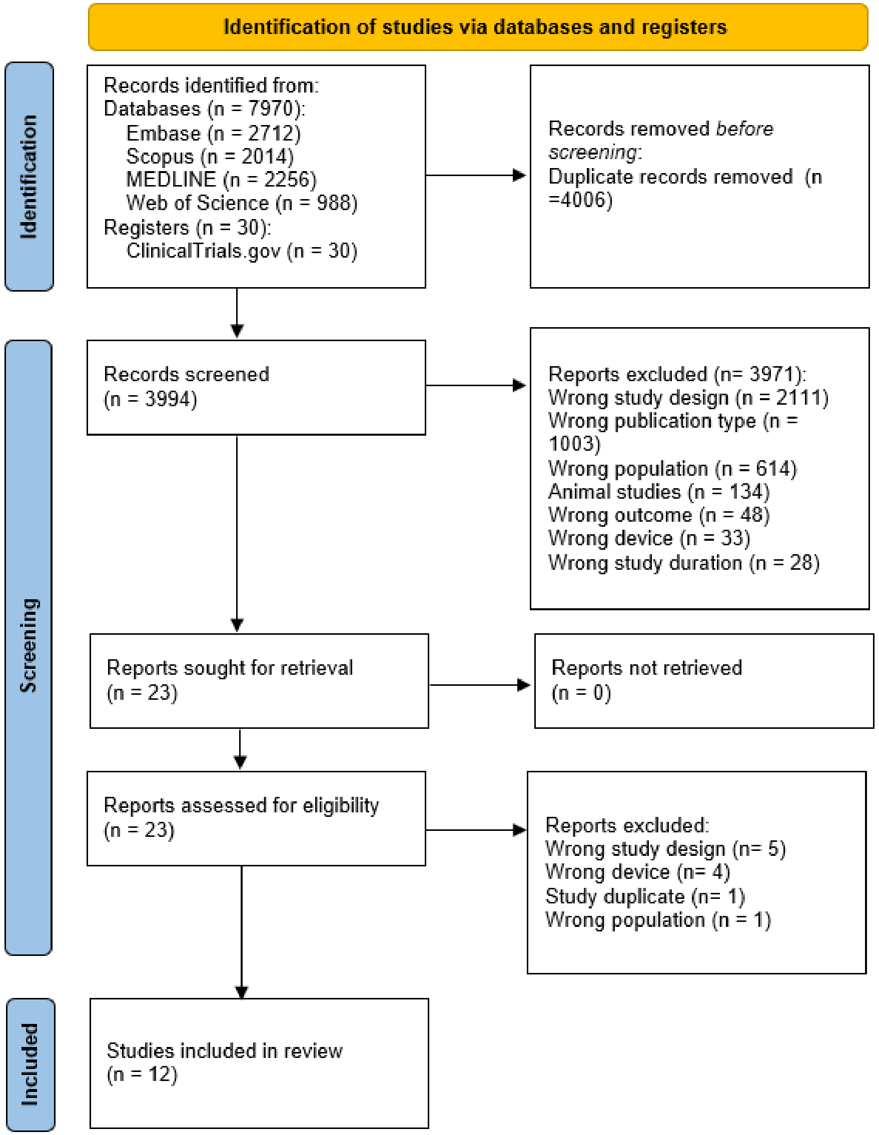

ESM Fig. 2 Risk of bias in the included studies

Intention-to-treat

Unique ID	Study ID	Experimental	Comparator	Outcome	Weight	D1	D2	D3	D4	D5	Overall
A	Ajjan 2023	isCGM	SMBG	HbA1c	1	+	+	+	+	+	+
B	Beck 2017	rtCGM	SMBG	HbA1c	1	+	+	+	+	+	+
C	Bergenstal 2022	rtCGM	SMBG	HbA1c	1	+	+	+	+	!	!
D	Cosson 2009	rtCGM	SMBG	HbA1c	1	+	+	+	+	!	!
E	Haak 2017	isCGM	SMBG	HbA1c	1	+	+	+	+	!	!
F	Martens 2021	rtCGM	SMBG	HbA1c	1	+	+	+	+	+	+
G	Moon 2022	rtCGM	SMBG	HbA1c	1	+	+	+	+	!	!
H	Price 2021	rtCGM	SMBG	HbA1c	1	!	+	+	+	!	!
I	Vigersky 2012	rtCGM	SMBG	HbA1c	1	!	+	+	+	+	!
J	Wada 2020	isCGM	SMBG	HbA1c	1	+	+	+	+	+	+
K	Yaron 2019	isCGM	SMBG	HbA1c	1	!	+	+	+	!	!
L	Yoo 2008	rtCGM	SMBG	HbA1c	1	+	+	+	+	!	!

+ Low risk
! Some concerns
- High risk

D1 Randomisation process
D2 Deviations from the intended interventions
D3 Missing outcome data
D4 Measurement of the outcome
D5 Selection of the reported result

ESM Fig. 3 Funnel plot of the main analysis (change in HbA1c).

MD: Mean difference. SE: Standard error.

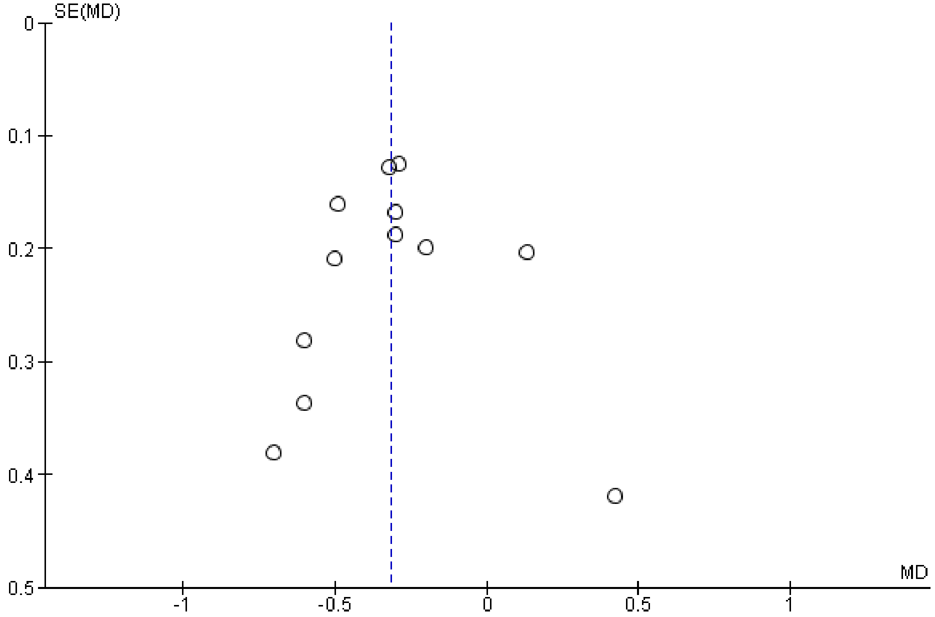

ESM Fig. 4 Forest plot of change in HbA1c (mmol/mol) in individuals with type 2 diabetes using real time or intermittently scanning continuous glucose monitoring compared to self-monitoring of blood glucose; results according to median baseline HbA1c (69 mmol/mol).

	CGM			SMBG				Mean Difference
**Study or Subgroup**	**Mean**	**SD**	**Total**	**Mean**	**SD**	**Total**	**Weight**	**IV, Random, 95% CI**
**1.12.1 HbA1c > 69 mmol/mol**								
Ajjan 2023	-5.36	21.3	55	-9.95	26.5	55	2.1%	4.59 [-4.40, 13.58]
Martens 2021	-12	16.4	116	-6.56	13.1	59	7.5%	-5.44 [-9.92, -0.96]
Yaron 2019	-8.96	9.18	53	-3.61	8.52	48	11.6%	-5.35 [-8.80, -1.90]
Beck 2017	-8.74	10.1	77	-5.46	12.5	75	10.7%	-3.28 [-6.90, 0.34]
Haak 2017	-3.06	14.2	149	-4.48	16.4	75	7.9%	1.42 [-2.94, 5.78]
Cosson 2009	-6.89	3.72	11	-3.39	3.17	14	16.3%	-3.50 [-6.25, -0.75]
Yoo 2008	-12	17.1	29	-4.37	14.2	28	2.5%	-7.63 [-15.78, 0.52]
**Subtotal (95% CI)**			**490**			**354**	**58.5%**	**-3.19 [-5.41, -0.97]**
Heterogeneity: Tau² = 3.71; Chi² = 10.80, df = 6 (P = 0.09); I² = 44%								
Test for overall effect: Z = 2.81 (P = 0.005)								
**1.12.2 HbA1c < 69 mmol/mol**								
Moon 2023	-6.56	7.65	15	0	12	15	3.2%	-6.56 [-13.76, 0.64]
Bergenstal 2022	-12.2	12	59	-8.96	9.84	55	9.0%	-3.24 [-7.26, 0.78]
Price 2021	-5.46	9.84	44	-3.28	7.65	23	8.1%	-2.18 [-6.45, 2.09]
Wada 2020	-5.03	5.14	48	-1.86	7.76	45	16.8%	-3.17 [-5.86, -0.48]
Vigersky 2012	-8.74	16.4	50	-2.19	14.2	50	4.4%	-6.55 [-12.56, -0.54]
**Subtotal (95% CI)**			**216**			**188**	**41.5%**	**-3.53 [-5.35, -1.71]**
Heterogeneity: Tau² = 0.00; Chi² = 2.12, df = 4 (P = 0.71); I² = 0%								
Test for overall effect: Z = 3.80 (P = 0.0001)								
**Total (95% CI)**			**706**			**542**	**100.0%**	**-3.43 [-4.75, -2.11]**
Heterogeneity: Tau² = 0.80; Chi² = 12.95, df = 11 (P = 0.30); I² = 15%								
Test for overall effect: Z = 5.11 (P < 0.00001)								
Test for subgroup differences: Chi² = 0.05, df = 1 (P = 0.82), I² = 0%								

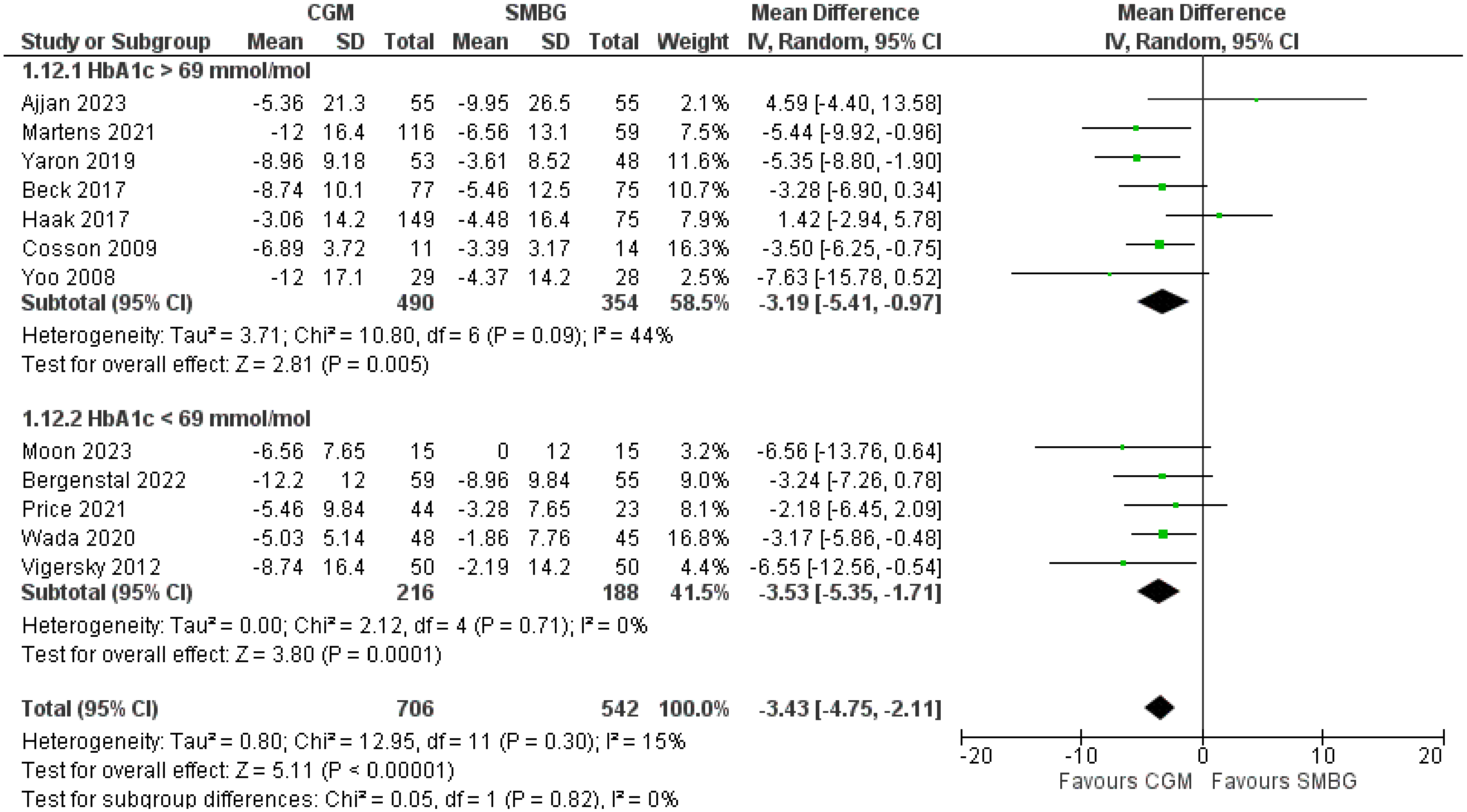

ESM Fig. 5 Forest plot of change in HbA1c (mmol/mol) in individuals with type 2 diabetes using real time or intermittently scanning continuous glucose monitoring compared to self-monitoring of blood glucose; results according to median baseline age (58.9 years).

	CGM			SMBG				Mean Difference
**Study or Subgroup**	**Mean**	**SD**	**Total**	**Mean**	**SD**	**Total**	**Weight**	**IV, Random, 95% CI**
**1.13.1 Age > 58.9 years**								
Ajjan 2023	-5.36	21.3	55	-9.95	26.5	55	2.1%	4.59 [-4.40, 13.58]
Bergenstal 2022	-12.2	12	59	-8.96	9.84	55	9.0%	-3.24 [-7.26, 0.78]
Price 2021	-5.46	9.84	44	-3.28	7.65	23	8.1%	-2.18 [-6.45, 2.09]
Yaron 2019	-8.96	9.18	53	-3.61	8.52	48	11.6%	-5.35 [-8.80, -1.90]
Beck 2017	-8.74	10.1	77	-5.46	12.5	75	10.7%	-3.28 [-6.90, 0.34]
Haak 2017	-3.06	14.2	149	-4.48	16.4	75	7.9%	1.42 [-2.94, 5.78]
**Subtotal (95% CI)**			**437**			**331**	**49.4%**	**-2.30 [-4.59, -0.01]**
Heterogeneity: Tau² = 3.24; Chi² = 8.44, df = 5 (P = 0.13); I² = 41%								
Test for overall effect: Z = 1.97 (P = 0.05)								
**1.13.2 Age < 58.9 years**								
Moon 2023	-6.56	7.65	15	0	12	15	3.2%	-6.56 [-13.76, 0.64]
Martens 2021	-12	16.4	116	-6.56	13.1	59	7.5%	-5.44 [-9.92, -0.96]
Wada 2020	-5.03	5.14	48	-1.86	7.76	45	16.8%	-3.17 [-5.86, -0.48]
Vigersky 2012	-8.74	16.4	50	-2.19	14.2	50	4.4%	-6.55 [-12.56, -0.54]
Cosson 2009	-6.89	3.72	11	-3.39	3.17	14	16.3%	-3.50 [-6.25, -0.75]
Yoo 2008	-12	17.1	29	-4.37	14.2	28	2.5%	-7.63 [-15.78, 0.52]
**Subtotal (95% CI)**			**269**			**211**	**50.6%**	**-4.17 [-5.79, -2.55]**
Heterogeneity: Tau² = 0.00; Chi² = 2.78, df = 5 (P = 0.73); I² = 0%								
Test for overall effect: Z = 5.05 (P < 0.00001)								
**Total (95% CI)**			**706**			**542**	**100.0%**	**-3.43 [-4.75, -2.11]**
Heterogeneity: Tau² = 0.80; Chi² = 12.95, df = 11 (P = 0.30); I² = 15%								
Test for overall effect: Z = 5.11 (P < 0.00001)								
Test for subgroup differences: Chi² = 1.71, df = 1 (P = 0.19), I² = 41.5%								

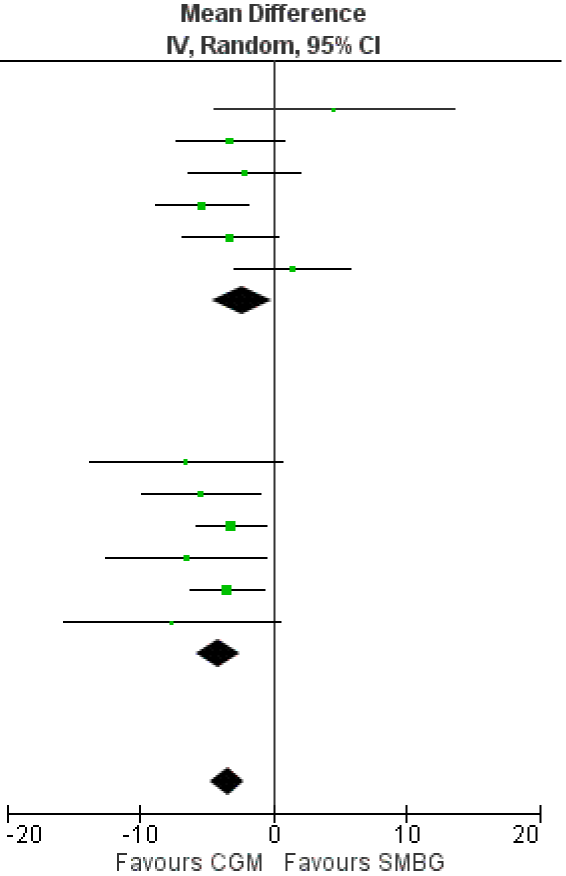

ESM Fig. 6 Forest plot of change in HbA1c (mmol/mol) in individuals with type 2 diabetes using real time or intermittently scanning continuous glucose monitoring compared to self-monitoring of blood glucose; results according to median diabetes duration (13.4 years).

	CGM			SMBG				Mean Difference
Study or Subgroup	Mean	SD	Total	Mean	SD	Total	Weight	IV, Random, 95% CI
**1.14.1 Diabetes duration > 13.4 years**								
Ajjan 2023	-5.36	21.3	55	-9.95	26.5	55	3.0%	4.59 [-4.40, 13.58]
Martens 2021	-12	16.4	116	-6.56	13.1	59	9.8%	-5.44 [-9.92, -0.96]
Yaron 2019	-8.96	9.18	53	-3.61	8.52	48	14.3%	-5.35 [-8.80, -1.90]
Beck 2017	-8.74	10.1	77	-5.46	12.5	75	13.5%	-3.28 [-6.90, 0.34]
Haak 2017	-3.06	14.2	149	-4.48	16.4	75	10.3%	1.42 [-2.94, 5.78]
**Subtotal (95% CI)**			**450**			**312**	**50.9%**	**-2.54 [-5.66, 0.58]**
Heterogeneity: Tau² = 7.03; Chi² = 9.64, df = 4 (P = 0.05); I² = 58%								
Test for overall effect: Z = 1.60 (P = 0.11)								
**1.14.2 Diabetes duration < 13.4 years**								
Moon 2023	-6.56	7.65	15	0	12	15	4.4%	-6.56 [-13.76, 0.64]
Bergenstal 2022	-12.2	12	59	-8.96	9.84	55	11.6%	-3.24 [-7.26, 0.78]
Price 2021	-5.46	9.84	44	-3.28	7.65	23	10.6%	-2.18 [-6.45, 2.09]
Cosson 2009	-6.89	3.72	11	-3.39	3.17	14	18.9%	-3.50 [-6.25, -0.75]
Yoo 2008	-12	17.1	29	-4.37	14.2	28	3.5%	-7.63 [-15.78, 0.52]
**Subtotal (95% CI)**			**158**			**135**	**49.1%**	**-3.62 [-5.50, -1.74]**
Heterogeneity: Tau² = 0.00; Chi² = 2.05, df = 4 (P = 0.73); I² = 0%								
Test for overall effect: Z = 3.77 (P = 0.0002)								
**Total (95% CI)**			**608**			**447**	**100.0%**	**-3.29 [-4.89, -1.69]**
Heterogeneity: Tau² = 1.55; Chi² = 11.86, df = 9 (P = 0.22); I² = 24%								
Test for overall effect: Z = 4.03 (P < 0.0001)								
Test for subgroup differences: Chi² = 0.34, df = 1 (P = 0.56), I² = 0%								

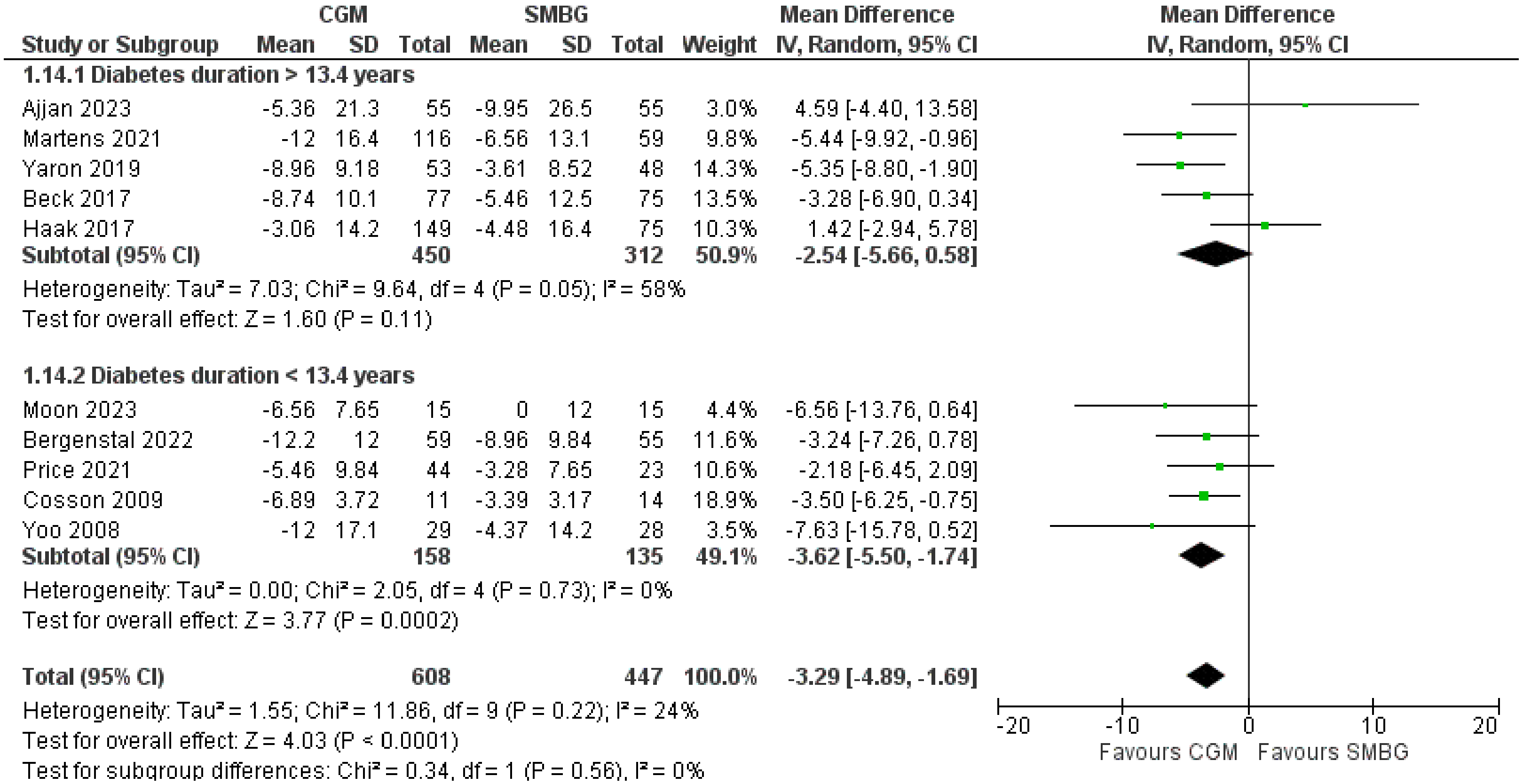

ESM Fig. 7 Forest plot of change in HbA1c (mmol/mol) in individuals with type 2 diabetes using real time or intermittently scanning continuous glucose monitoring compared to self-monitoring of blood glucose; results according to median intervention duration (12 weeks).

	CGM			SMBG				Mean Difference
**Study or Subgroup**	**Mean**	**SD**	**Total**	**Mean**	**SD**	**Total**	**Weight**	**IV, Random, 95% CI**
**1.10.1 Intervention duration ≤ 12 weeks**								
Ajjan 2023	-5.36	21.3	55	-9.95	26.5	55	2.4%	4.59 [-4.40, 13.58]
Moon 2023	-6.56	7.65	15	0	12	15	3.7%	-6.56 [-13.76, 0.64]
Wada 2020	-5.03	5.14	48	-1.86	7.76	45	17.5%	-3.17 [-5.86, -0.48]
Yaron 2019	-8.96	9.18	53	-3.61	8.52	48	12.5%	-5.35 [-8.80, -1.90]
Vigersky 2012	-8.74	16.4	50	-2.19	14.2	50	5.1%	-6.55 [-12.56, -0.54]
Cosson 2009	-6.89	3.72	11	-3.39	3.17	14	17.0%	-3.50 [-6.25, -0.75]
Yoo 2008	-12	17.1	29	-4.37	14.2	28	2.9%	-7.63 [-15.78, 0.52]
**Subtotal (95% CI)**			**261**			**255**	**61.2%**	**-4.04 [-5.70, -2.39]**
Heterogeneity: Tau² = 0.43; Chi² = 6.53, df = 6 (P = 0.37); I² = 8%								
Test for overall effect: Z = 4.80 (P < 0.00001)								
**1.10.2 Intervention duration > 12 weeks**								
Bergenstal 2022	-12.2	12	59	-8.96	9.84	55	10.0%	-3.24 [-7.26, 0.78]
Martens 2021	-12	16.4	116	-6.56	13.1	59	8.4%	-5.44 [-9.92, -0.96]
Beck 2017	-8.74	10.1	77	-5.46	12.5	75	11.7%	-3.28 [-6.90, 0.34]
Haak 2017	-3.06	14.2	149	-4.48	16.4	75	8.8%	1.42 [-2.94, 5.78]
**Subtotal (95% CI)**			**401**			**264**	**38.8%**	**-2.66 [-5.32, -0.01]**
Heterogeneity: Tau² = 2.97; Chi² = 5.04, df = 3 (P = 0.17); I² = 40%								
Test for overall effect: Z = 1.96 (P = 0.05)								
**Total (95% CI)**			**662**			**519**	**100.0%**	**-3.54 [-4.98, -2.11]**
Heterogeneity: Tau² = 1.17; Chi² = 12.59, df = 10 (P = 0.25); I² = 21%								
Test for overall effect: Z = 4.84 (P < 0.00001)								
Test for subgroup differences: Chi² = 0.75, df = 1 (P = 0.39), I² = 0%								

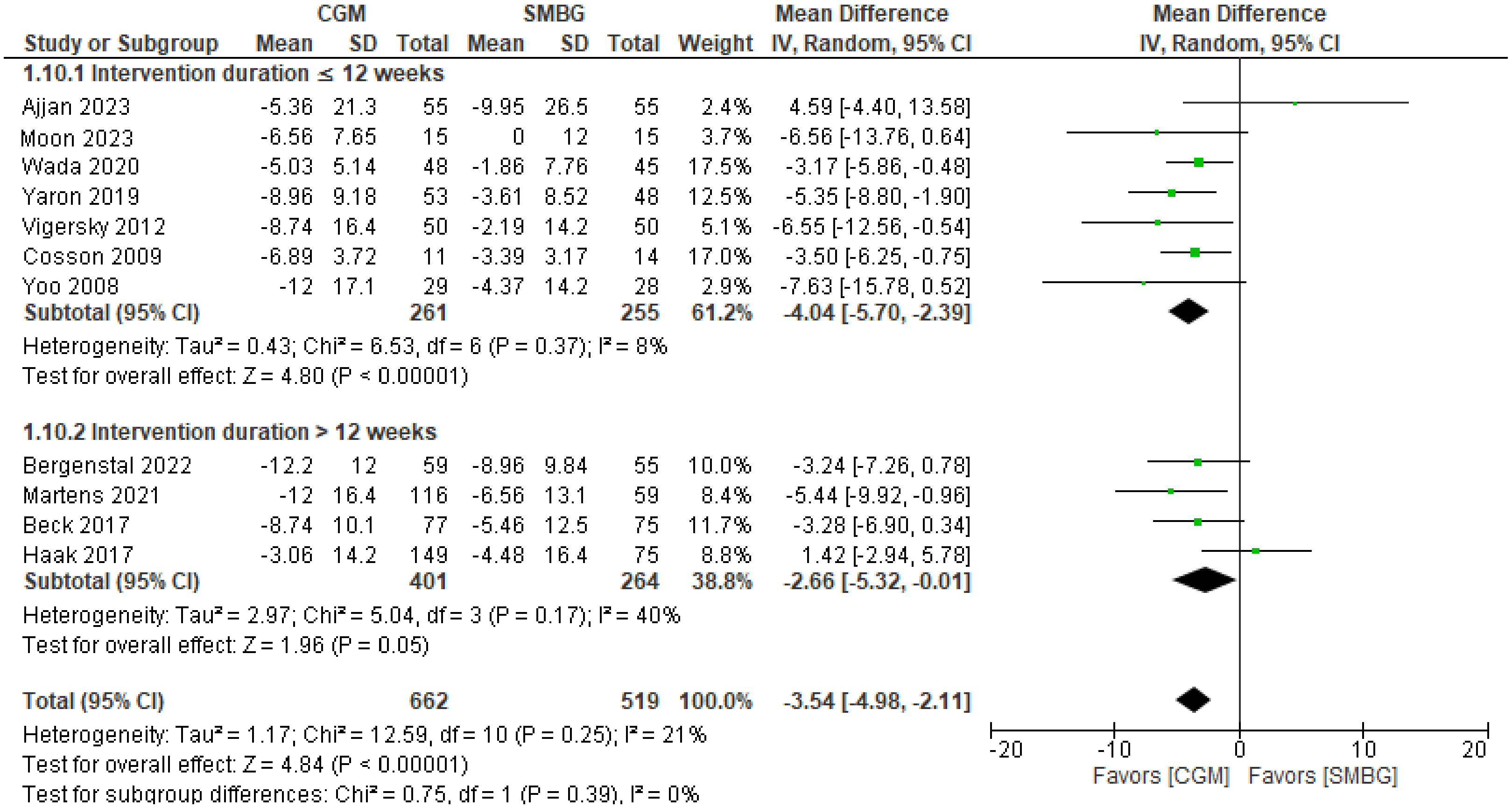

ESM Fig. 8 Forest plot of change in glycemic variability (coefficient of variation (%)) in individuals with type 2 diabetes using real time or intermittently scanning continuous glucose monitoring compared to self-monitoring of blood glucose.

	CGM			SMBG				Mean Difference
**Study or Subgroup**	**Mean**	**SD**	**Total**	**Mean**	**SD**	**Total**	**Weight**	**IV, Random, 95% CI**
Moon 2023	-0.8	7.9	15	0.1	5.1	15	9.5%	-0.90 [-5.66, 3.86]
Martens 2021	-0.4	9.22	116	1	9.22	59	25.7%	-1.40 [-4.29, 1.49]
Wada 2020	-0.3	8.44	41	-1	7.8	35	16.1%	0.70 [-2.95, 4.35]
Beck 2017	-2	9.98	78	0	9.93	75	21.6%	-2.00 [-5.16, 1.16]
Haak 2017	-2.7	9.5	149	-0.1	10.43	75	27.2%	-2.60 [-5.41, 0.21]
**Total (95% CI)**			**399**			**259**	**100.0%**	**-1.47 [-2.94, -0.01]**

Heterogeneity: Tau² = 0.00; Chi² = 2.14, df = 4 (P = 0.71); I² = 0%
Test for overall effect: Z = 1.97 (P = 0.05)

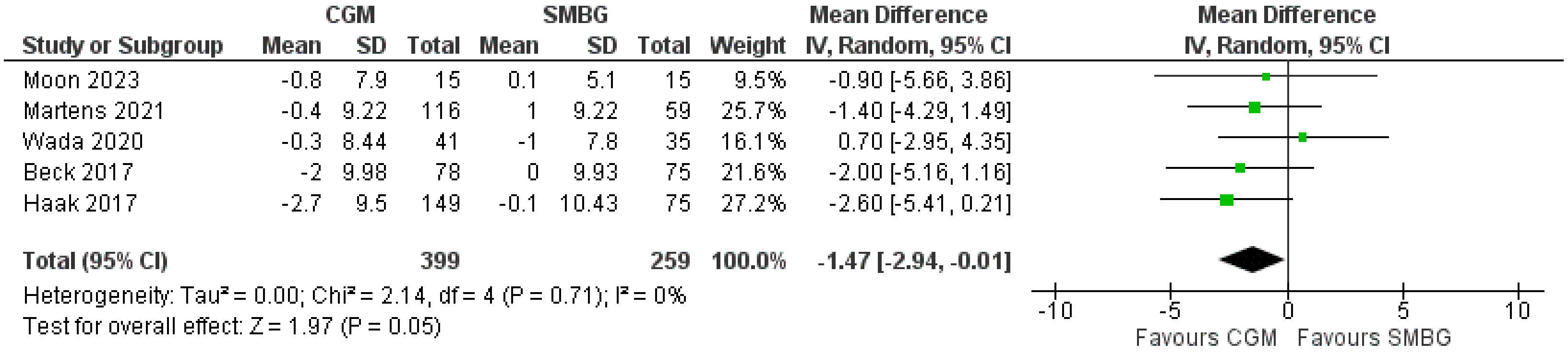

ESM Fig. 9 Forest plot of change in the incident risk of severe hypoglycemia in individuals with type 2 diabetes using real time or intermittently scanning continuous glucose monitoring compared to self-monitoring of blood glucose.

	CGM		SMBG			Risk Ratio
**Study or Subgroup**	**Events**	**Total**	**Events**	**Total**	**Weight**	**M-H, Random, 95% CI**
Ajjan 2023	0	69	2	72	25.0%	0.21 [0.01, 4.27]
Martens 2021	1	116	1	59	30.0%	0.51 [0.03, 7.99]
Haak 2017	3	149	1	75	45.1%	1.51 [0.16, 14.27]
**Total (95% CI)**		**334**		**206**	**100.0%**	**0.66 [0.15, 3.00]**
Total events	4		4			

Heterogeneity: Tau² = 0.00; Chi² = 1.12, df = 2 (P = 0.57); I² = 0%
Test for overall effect: Z = 0.53 (P = 0.60)

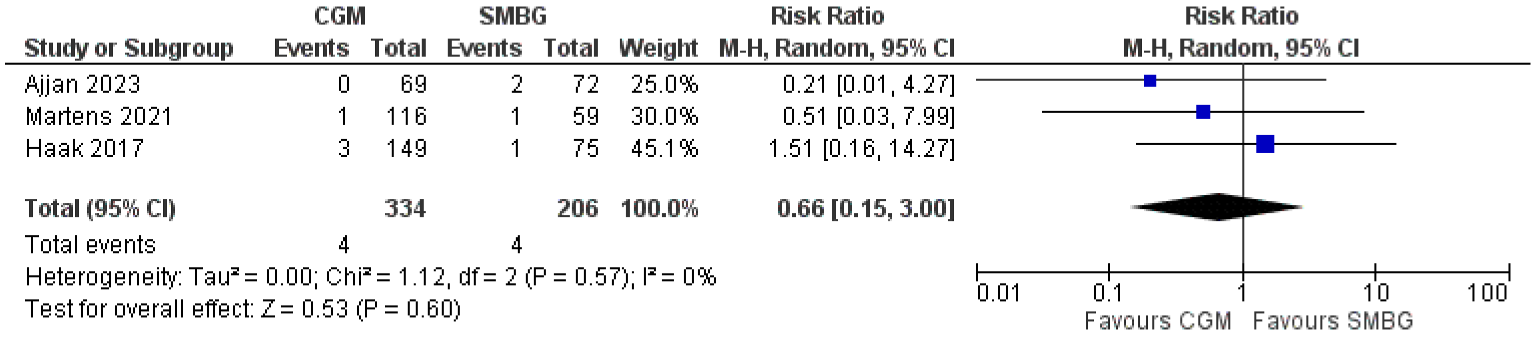

ESM Fig. 10 Forest plot of change in the incident risk on macrovascular complications in individuals with type 2 diabetes using real time or intermittently scanning continuous glucose monitoring compared to self-monitoring of blood glucose.

	CGM		SMBG			Risk Ratio
**Study or Subgroup**	**Events**	**Total**	**Events**	**Total**	**Weight**	**M-H, Random, 95% CI**
Ajjan 2023	18	69	11	72	69.6%	1.71 [0.87, 3.35]
Price 2021	0	44	1	23	14.3%	0.18 [0.01, 4.20]
Beck 2017	3	77	0	75	16.1%	6.82 [0.36, 129.83]
**Total (95% CI)**		**190**		**170**	**100.0%**	**1.54 [0.42, 5.72]**
Total events	21		12			

Heterogeneity: Tau² = 0.52; Chi² = 2.81, df = 2 (P = 0.25); I² = 29%

Test for overall effect: Z = 0.65 (P = 0.52)

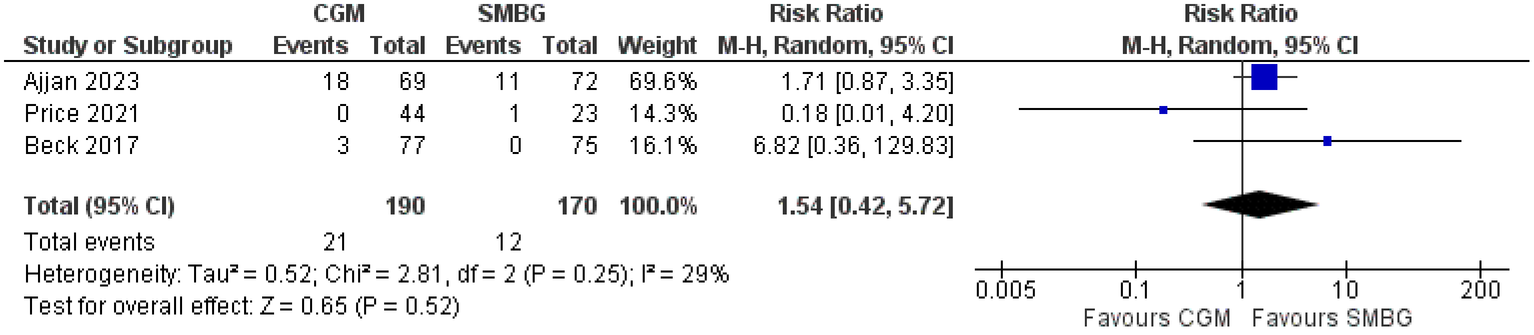

Supplement: Supplementary file 1 — Supplementary file1 (PDF 337 KB) [file 125_2024_6107_MOESM1_ESM.pdf]
